# Secondary Traumatic Stress in Obstetrics and Gynecology Faculty, House Officers, and Certified Nurse Midwives

**DOI:** 10.1177/26884844251394212

**Published:** 2025-11-04

**Authors:** Elizabeth Campbell, Helen K. Morgan, James T. Fitzgerald, Angela Liang

**Affiliations:** ^1^ Department of Obstetrics and Gynecology, University of Michigan, Ann Arbor, Michigan, USA.; ^2^ Department of Learning Health Sciences, University of Michigan, Ann Arbor, Michigan, USA.

**Keywords:** certified nurse midwives, house officers, Obstetrics and gynecology faculty, peer support, second victim, secondary traumatic stress

## Abstract

**Background:**

Although the prevalence of secondary traumatic stress (STS) in Obstetrics and Gynecology (OBGYN) is established, little is known about its effect on different OBGYN providers. We examined variances in impact of STS between OBGYN faculty, house officers (HO), and certified nurse midwives (CNM), as well as barriers and desired resources for support.

**Methods:**

All OBGYN providers at one academic institution received a survey in May 2024 assessing prevalence of STS, distress, resources, and barriers to support. Differences among provider types were examined by likelihood-ratio Chi-squared tests.

**Results:**

Of 165 individuals receiving the survey, 92 (55.8%) responded (faculty 57.0%, HOs 57.8%, CNMs 50.0%). Most respondents (91.3%) had experienced a traumatic work event; faculty reported the highest rate (95.9%). While all respondents experienced high rates of psychological and physical distress from an adverse event, faculty were most fearful of future occurrences (90.9% vs. HOs 69.6%, CNMs 53.9%, *p* = 0.03) and reported more difficulty sleeping (36.4% vs. HOs 17.4%, CNMs 23.1%, *p* = 0.04). Adverse events negatively impacted professional self-efficacy. Faculty, particularly, reported high rates (76%) of feeling inadequate, although this was not statistically different from HOs (55%) or CNMs (50%), *p* = 0.31. All groups cited involvement in a legal situation as the principal reason for seeking support, peer support as the preferred intervention, and time as the biggest barrier to seeking support.

**Conclusions:**

Nearly all OBGYN providers experience STS after adverse events. This study highlights stressors unique to faculty. Intentional programming and support for faculty are needed to promote well-being and professional development.

## Introduction

When medical errors or adverse events occur, harm extends well beyond the patient. The term “second victim” reflects the impact on health care professionals when mistakes occur.^1^ Second victims are at risk to develop secondary traumatic stress (STS), a form of post-traumatic stress resulting from direct or indirect involvement in an adverse event.^2^ Failure to address emotional and physical symptoms for those who develop STS can impact overall wellness.^2^ Providers with STS commonly report symptoms of anxiety, guilt, and disrupted sleep. There are significant consequences to health care providers when STS is not addressed, which may ultimately lead to burnout.^3^ The effects of adverse outcomes also extend to the well-being of learners. Following adverse outcomes, medical students and resident physicians can develop increasing feelings of guilt, anger, and fear.^4^

Across different specialties, second victim phenomenon prevalence rates vary depending on the population sampled, ranging from 10% to almost 75%.^5^ Existing studies suggest a particularly high incidence of adverse events and STS among Obstetrics and Gynecology (OBGYN) supervising physicians and house officers (HOs). Up to 90% of OBGYN providers have been involved in adverse medical events, with the majority of providers identifying as a second victim and up to 75% developing signs of traumatic stress.^2,6^ Similarly, certified nurse midwives (CNMs) experience high incidences of STS, with up to a third of midwives developing post-traumatic stress symptoms.^7^ In a 2012 national survey of all Danish obstetricians and midwives, obstetricians had experienced more traumatic births than midwives (median 3 vs. 2); however, with the exception of general stress, midwives had higher levels of burnout, sleep disorders, depression symptoms, somatic stress, and cognitive stress.^8^ Similarly, a survey in Spain demonstrated that obstetricians had greater dimensions of physical distress and absenteeism, while midwives had higher dimensions of psychological distress and more negative impact to professional self-efficacy.^9^ When OBGYN providers—including faculty physicians, HOs, and CNMs—have been queried about support following adverse outcomes, the majority desired formalized support at a departmental or institutional level, especially from peer-to-peer responders.^2,9–11^

Although the high prevalence of STS is established in OBGYN, with studies existing for physicians, trainees, and CNMs individually, there is a paucity of data both examining how STS differentially impacts each group and directly comparing experiences and differences between the groups. Depending on the practice, the three groups may have varying degrees of responsibilities within the framework of a health system. OBGYN HOs are, by definition, trainees who provide patient care under the supervision of faculty OBGYN physicians. For CNMs, there is significant variation in practice patterns, with collaboration with physicians existing on a continuum. For example, CNMs may manage an independent practice, be employed by a physician-owned practice, or work within a health system or institution that hires both midwives and physicians—all of which may have differing levels of physician collaboration.^12^ To our knowledge, no U.S. studies have evaluated differences in traumatic stress between OBGYN physicians/trainees and midwives. And while peer support has been identified for the groups as a useful resource after adverse outcomes, comparisons of desired resources between the groups has not been studied explicitly.

The objective of this study was to assess the impact and resources desired for STS and assess for differences between OBGYN faculty, trainees, and CNMs at a single institution.

## Materials and Methods

This study was conducted within the department of OBGYN at a single large academic medical center. An online survey was created based on a review of the literature using questions from the Second Victim Experience and Support Tool (SVEST)^2^ and Hu et al.^13^ SVEST is a survey with validity evidence that evaluates second victim experiences of staff, as well as quality of support resources. Survey questions focusing on motivations and barriers to seeking support were drawn from Hu et al.^13^

The survey was distributed by email to all clinical faculty, residents, fellows, and CNMs in May 2024, with a second email sent as a reminder in June 2024. The first 150 respondents were offered a $20 gift card as an incentive for completing the survey. Responses were collected anonymously. The study was deemed exempt by the University of Michigan Institutional Review Board (HUM00221059). Participant consent was indicated by survey continuation.

Survey question responses on a 5-point Likert scale were categorized into three groups: “agree” (strongly agree and agree); “neutral”; and “disagree” (strongly disagree and disagree). Frequency tables were used to determine response rates and overall descriptive statistics. Differences among faculty, HOs, and CNMs were examined by likelihood-ratio Chi-squared tests.

## Results

The survey was sent to a total of 165 individuals (86 faculty, 45 HOs, and 34 CNMs). Overall, we received responses from 92/165 (55.8%) individuals, with response rates of 49/86 (57.0%) for faculty, 26/45 (57.8%) for HOs, and 17/34 (50.0%) for CNMs. The majority of respondents (91.3%, 84/92) reported having experienced a traumatic work event. Nearly all faculty (95.9%, 47/49) and HOs (92.3%, 24/26) reported experiencing an adverse event, compared with 76.5% (13/17) for CNMs (*p* = 0.078). Less than half of all respondents (43.8%, 35/80) reported feeling supported when going through an adverse professional event: faculty 37.2% (16/43), HOs 50.0% (12/24), and CNMs 53.9% (7/13).

Faculty, HOs, and CNMs indicated high rates of psychological and physical distress in response to an adverse event. Results are presented in Table 1. Notably, nearly all faculty (90.9%, 40/44) reported feeling fearful of future occurrences and were more likely than other groups to indicate this (*p* = 0.03). All providers reported high levels of psychological distress (embarrassment, feeling miserable, and feeling remorse), with no differences between the three groups. On measures of physical distress, 36.4% (16/44) of faculty experienced difficulty sleeping as a result of their involvement in traumatic work events (*p* = 0.04). Incidences of exhaustion, feeling queasy or nauseous, and poor appetite were not statistically different between groups.

**Table 1.** Second Victim Experience and Support Tool dimensions of psychological and physical distress and professional self-efficacy

Element	Faculty (*n* = 50)	House officers (*n* = 26)	CNMs (*n* = 18)	*p* value^a^
Psychological distress				
Embarrassment^b^	30 (68.2)	11 (47.8)	8 (61.5)	.52
Fearful of future occurrences^b^	40 (90.9)	16 (60.6)	7 (53.9)	.03
Feeling miserable^c^	30 (68.2)	12 (52.2)	11 (91.7)	.21
Feeling remorse^c^	26 (59.0)	12 (52.2)	6 (50.0)	.71
Physical distress				
Mental weight exhausting^b^	32 (72.7)	12 (52.2)	7 (53.9)	.41
Difficulty sleeping^b^	16 (36.4)	4 (17.4)	13 (23.1)	.04
Feel queasy or nauseous^b^	17 (38.6)	9 (39.1)	6 (46.2)	.22
Poor appetite^b^	11 (25.0)	6 (26.1)	5 (38.5)	.63
Professional self-efficacy				
Feelings of inadequacy regarding patient care abilities^d^	32 (76.0)	12 (55.5)	6 (50.0)	.31
Makes me wonder if I’m not really a good health care provider^d^	28 (66.7)	9 (40.9)	6 (50.0)	.32
Became afraid to attempt difficult or high-risk procedures^d^	28 (66.7)	9 (40.9)	5 (41.7)	.11
Don’t make me question my professional abilities^d^	7 (16.7)	20 (26.3)	12 (33.3)	.28

Data presented as *n* (%).

^a^ Likelihood-ratio Chi-squared test

^b^ Responses based on Faculty *n* = 44, House officers *n* = 23, CNMs *n* = 13.

^c^ Responses based on Faculty *n* = 44, House officers *n* = 23, CNMs *n* = 12.

^d^ Responses based on Faculty *n* = 42, House officers *n* = 22, CNMs *n* = 12.

CNM, certified nurse-midwife.

Adverse patient care events had a substantial negative impact on sense of professional self-efficacy for faculty, HOs, and CNMs. While none reached statistical significance, faculty had higher scores on all metrics (Table 1). More than half of all respondents expressed feelings of inadequacy regarding patient care (faculty 76.2%, HOs 55.5%, CNMs 50.0%). The majority of faculty (66.7%, 28/42) reported that these events make them question whether they are a good health care provider and fearful of attempting difficult procedures in the future.

Involvement in a legal situation was the reason for seeking support most cited by survey participants overall (84.3%, 70/83). When responses were evaluated by role, CNMs were the only group for which this remained the most likely, while substance abuse and physical illness were most often cited by faculty and HOs, respectively (Table 2). Notably, less than half of participants responded that they would seek support for interpersonal conflict or burnout. Respondents were least likely to seek support for fatigue (19.3% overall, 16/83).

**Table 2.** Reasons for seeking support

Difficulty	Faculty (*n* = 50)	House officers (*n* = 26)	CNMs (*n* = 18)	Overall (*N* = 94)	*p* value^a^
Fatigue^b^	6 (14.0)	9 (37.5)	1 (6.3)	16 (19.3)	0.07
Interpersonal conflict^b^	13 (30.2)	9 (37.5)	6 (37.5)	28 (33.7)	0.61
Personal burnout^b^	20 (46.5)	12 (50.0)	8 (50.0)	40 (48.2)	0.69
Mental illness in family^b^	17 (39.5)	7 (29.2)	9 (56.3)	33 (39.8)	0.52
Personal life struggles^c^	20 (47.6)	10 (41.7)	7 (43.8)	37 (45.1)	0.56
Physical illness in family^c^	25 (59.5)	14 (58.3)	7 (43.8)	46 (56.1)	0.66
Poor patient outcomes^c^	20 (47.6)	16 (66.7)	8 (50.0)	44 (53.7)	0.38
Mental illness in self^c^	34 (81.0)	18 (75.0)	12 (75.0)	64 (78.0)	0.68
Interpersonal conflict in workplace^c^	18 (42.9)	18 (75.0)	3 (18.8)	39 (47.6)	0.14
Physical illness in self^c^	33 (78.6)	22 (91.7)	11 (68.8)	55 (80.5)	0.43
Involved in adverse event^c^	19 (45.2)	15 (62.5)	11 (68.8)	45 (54.9)	0.19
Substance abuse^c^	36 (85.7)	18 (75.0)	12 (75.0)	66 (80.5)	0.83
Involved in medical error^d^	25 (58.1)	18 (75.0)	11 (73.3)	54 (65.9)	0.34
Legal situation^b^	34 (79.0)	21 (87.5)	15 (93.8)	70 (84.3)	0.57

Data presented as *n* (%).

^a^ Likelihood-ratio Chi-squared test.

^b^ Responses based on Faculty *n* = 43, House officers *n* = 24, CNMs *n* = 16, Overall *n* = 83.

^c^ Responses based on Faculty *n* = 42, House officers *n* = 24, CNMs *n* = 16, Overall *n* = 82.

^d^ Responses based on Faculty *n* = 43, House officers *n* = 24, CNMs *n* = 15, Overall *n* = 82.

CNM, certified nurse-midwife.

In response to the desirability of proposed interventions, all groups selected peer-to-peer support as their preferred method (62/94, 66% of respondents overall). Emotional debriefing sessions and departmental culture were the other preferred interventions identified by faculty and CNMs, while HO selections were more evenly distributed among all options (Table 3). All groups cited lack of time as the biggest barrier to seeking support (faculty 88.4% [38/43], HOs 95.8% [23/24], CNMs 81.3% [13/16], *p* = 0.25). Additional barriers and participant responses are displayed in Table 4.

**Table 3.** Desirability of support options

Support option	Faculty (*n* = 50)	House officers (*n* = 26)	CNMs (*n* = 18)	*p* value^a^
Peer-to-peer support	32 (64.0)	17 (65.4)	13 (72.2)	0.81
Emotional debriefing session	24 (48.0)	7 (26.9)	11 (61.1)	0.06
Mental health professional	17 (34.0)	7 (26.9)	9 (50.0)	0.29
Ombudsman	1 (2.0)	4 (15.4)	0	0.03
Department culture	22 (44.0)	11 (42.3)	7 (38.9)	0.93
Nonwork environment socializing with colleagues	15 (30.0)	7 (26.9)	4 (22.2)	0.81
Other	1 (2.0)	0	0	0.53
Do not desire programs	0	0	0	—

Data presented as *n* (%).

^a^ Likelihood-ratio Chi-squared test.

CNM, certified nurse-midwife.

**Table 4.** Factors that would be a barrier to seeking support

Factor	Faculty (*n* = 50)	House officers (*n* = 26)	CNMs (*n* = 18)	*p* value^a^
Nobody will understand^b^	8 (18.6)	5 (20.8)	5 (31.3)	0.33
My problems are not important^b^	10 (23.3)	6 (25.0)	7 (43.8)	0.30
Cost^b^	9 (20.9)	9 (37.5)	4 (25.0)	0.29
Using services means I am weak^c^	9 (24.3)	1 (4.8)	2 (15.4)	0.12
Fear of legal consequences^b^	15 (34.9)	9 (37.5)	3 (18.8)	0.12
Fear of unwanted intervention^d^	22 (52.4)	14 (58.3)	7 (43.8)	0.74
Difficulty accessing services^b^	25 (58.1)	9 (37.5)	6 (37.5)	0.12
Not knowing who to go to^b^	25 (58.1)	23 (50.0)	9 (56.3)	0.75
Stigma of mental health care^b^	18 (41.9)	7 (29.2)	3 (18.8)	0.41
Fear of documentation on my record^e^	26 (60.5)	12 (52.2)	7 (43.8)	0.08
Fear of negative impact on my career^b^	26 (60.5)	12 (50.0)	5 (31.3)	0.26
Lack of confidentiality^b^	24 (55.8)	11 (45.8)	7 (43.8)	0.27
Lack of time^b^	38 (88.4)	23 (95.8)	13 (81.3)	0.29

Data presented as *n* (%).

^a^ Likelihood-ratio Chi-squared test.

^b^ Responses based on Faculty *n* = 43, House officers *n* = 24, CNMs *n* = 16.

^c^ Responses based on Faculty *n* = 37, House officers *n* = 21, CNMs *n* = 13.

^d^ Responses based on Faculty *n* = 42, House officers *n* = 24, CNMs *n* = 16.

^e^ Responses based on Faculty *n* = 43, House officers *n* = 23, CNMs *n* = 16.

CNM, certified nurse-midwife.

## Discussion

In this snapshot survey of OBGYN faculty, HOs, and CNMs at one institution, we found a high prevalence of physical and psychological distress resulting from adverse medical events. We identified key gaps that are needed to optimally support OBGYN health care providers, and in particular, unique needs for faculty physicians, who endorsed notably high levels of psychological and physical distress. Ultimately, faculty in our health system are responsible for patient care provided by both HOs and CNMs on labor and delivery, which may correspond with increased feelings of fear and difficulty sleeping for faculty.

These findings add to the growing body of literature around secondary trauma among health care providers, and to our knowledge, this is one of the only studies in the US that has identified differences in reactions to adverse events—particularly for faculty physicians. The National Academy of Medicine describes a wide variety of external and internal factors that influence clinician well-being, including the importance of organizational support and a positive learning and practice environment.^14^ This work highlights the need for departmental and organizational leadership to develop intentional support for faculty through peer support.

As seen in other studies, our providers expressed a desire for support in the workplace, particularly from their peers. More work is needed to determine how to optimize professional peer support programs. Existing programs have been developed for entire hospital systems, as well as for individual departments, with literature describing their programmatic design, recruitment of peer supporters, implementation and impact.^15–17^ While existing programs have been successful in implementation, they are not available for the majority of providers in the United States.^17^ Specialty-specific programs have been developed in surgery, psychiatry, and anesthesia; these are particularly successful because same-specialty colleagues have an improved ability to validate experiences given a shared background with similar circumstances.^16^ Importantly, peer support programs specifically targeting OBGYN practitioners have not been described in the literature—despite our study corroborating prior findings of high rates of adverse events, high prevalence of second victim experience among providers, and desire for such peer support programs.^2,6,11^ Future development of peer support programs created by OBGYNs for obstetric providers (including HOs, CNMs, and faculty) would help address this gap.

Notably, of all procedural specialties, OBGYN has been predominantly female for decades—currently, 85% of OBGYN residents in the United States identify as female.^18^ Female surgeons may personalize adverse events more than male surgeons,^19^ and have been shown to be at increased risk of developing STS, highlighting the impact gender may have on psychosocial well-being.^20,21^ Within our department, 84.9% (73/86) of faculty, 82.2% (37/45) of HOs, and 100% (34/34) of CNMs identify as female. We did not ask specifically about respondents’ gender due to the need to protect their anonymity given the low number of male HOs and faculty in our department. However, our findings point to the need for future work to determine whether there are differences in psychological and physical responses to secondary trauma between genders. Additional work is also needed to examine what interventions are able to reduce physical and psychological burden after a traumatic event. Although the prevalence of these types of events can not likely be reduced, it is imperative that effective interventions for when they do happen be implemented and examined.

This study is unique in the field of secondary trauma in that our study design allowed for comparison between different professional roles using a survey with prior validity evidence. Although prior studies have indicated a high incidence of traumatic stress in OBGYN providers individually, none to our knowledge have assessed for differences between faculty and trainees.

One limitation of the study is that our results may reflect a nonresponse bias, with those choosing to participate being more likely to have experienced or been negatively impacted by a traumatic event. In addition, our study was performed at a single institution and thus may not be generalizable to other clinical settings.

## Conclusion

Intentional supports are needed for OBGYN health care providers in the context of adverse medical outcomes, especially for faculty.

## Abbreviations Used

CNM: Certified nurse midwife
HO: House officers
OBGYN: Obstetrics and gynecology
STS: Secondary traumatic stress
